# Retrospect: The Outbreak Evaluation of COVID-19 in Wuhan District of China

**DOI:** 10.3390/healthcare9010061

**Published:** 2021-01-08

**Authors:** Yimin Zhou, Zuguo Chen, Xiangdong Wu, Zengwu Tian, Lingjian Ye, Leyi Zheng

**Affiliations:** 1 Shenzhen Institutes of Advanced Technology, Chinese Academy of Sciences, 1068 Xueyuan Avenue, Xili University Town, Shenzhen 518055, China; zg.chen1@siat.ac.cn (Z.C.); xd.wu1@siat.ac.cn (X.W.); zw.tian@siat.ac.cn (Z.T.); lj.ye@siat.ac.cn (L.Y.); ly.zheng@siat.ac.cn (L.Z.); 2 School of Computer Science and Technology, The University of Chinese Academy of Sciences, Beijing 100049, China; 3 School of Information and Electrical Engineering, Hunan University of Science and Technology, Xiangtan 411201, China

**Keywords:** COVID-19, C-SEIR, infection data, prevention measures

## Abstract

There were 27 novel coronavirus pneumonia cases found in Wuhan, China in December 2019, named as 2019-nCoV temporarily and COVID-19 formally by the World Health Organization (WHO) on the 11 February 2020. In December 2019 and January 2020, COVID-19 has spread on a large scale among the population, which brought terrible disaster to the life and property of the Chinese people. In this paper, we analyze the features and pattern of the virus transmission. Considering the influence of indirect transmission, a conscious-based Susceptible-Exposed-Infective-Recovered (SEIR) (C-SEIR) model is proposed, and the difference equation is used to establish the model. We simulated the C-SEIR model and key important parameters. The results show that (1) increasing people’s awareness of the virus can effectively reduce the spread of the virus; (2) as the capability and possibility of indirect infection increases, the proportion of people being infected will also increase; (3) the increased cure rate can effectively reduce the number of infected people. Then, the virus transmission can be modelled and used for the inflexion and extinction period of pandemic development so as to provide theoretical support for the Chinese government in the decision-making of pandemic prevention and recovery of economic production. Further, this study has demonstrated the effectiveness of the prevention measures taken by the Chinese government such as multi-level administrative district isolation and public health awareness.

## 1. Introduction

The outbreak of novel coronavirus pneumonia (NCP) in Wuhan at the end of 2019 has been spreading rapidly, which was designated as “public health emergencies of international concern” by World Health Organization (WHO). In just one month from the beginning of January 2020, COVID-19 has spread to more than 30 Provinces and Municipalities in China from the center of Wuhan [1]. This outbreak not only poses a huge threat to people’s health, but also has huge economic and political damage, i.e., changing our daily life [2].

In order to control the pandemic, Wuhan (Capital of Hubei Province) first announced closing of the city on the 24 January 2020 (Eve of Chinese New Year), then other Provinces and cities have started the first-class response to this major public health emergency in succession to control population flow [3]. At the same time, medical teams carrying a large amount of medical supplies from other Provinces of China have arrived in Wuhan immediately to support Wuhan local hospitals. However, the national pandemic situation, especially the situation in Hubei Province, is still disconcerting. The public is quite concerned about the development trend of the pandemic and expects the arrival of an “inflection point” [4].

The outbreak of COVID-19 has given rise to alarm for public health emergencies. So far, many medical problems about COVID-19 and its spread have yet to be fully studied and understood, and questions and lessons are worthy of investigating. Moreover, at the end of the Spring Festival holiday, many enterprises will be back to work and the possibility of re-emergence cannot be ignored and should be taken seriously. Therefore, it is necessary to further study the pandemic process and the trend of the development of COVID-19, and provide a strong basis for effective control of disease pandemics. One of the important research methods is to establish mathematical models for infectious diseases, and analyzes the properties of the related mathematical models, so as to reflect the laws of infectious diseases. The analysis before the establishment of the pandemic model is particularly important, since the spread of infectious diseases depends on the mechanism of the disease. The infection status of COVID-19 has a latent period, and COVID-19 can be indirectly infected, that is, through the treatment of COVID-19 medical waste, objects contacted by the infected person or close contact with the infected ones. Considering these factors, many people have used the classic Susceptible-Exposed-Infective-Recovered (SEIR) model to study COVID-19. Piasecki et al. established a SEIRQ model and studied the impact of isolation on the pandemic situation in Poland [5]. Yang et al. considered the inflow and outflow of population based on the classic SEIR model. Through the simulation of the model and the use of an artificial intelligence method, they verified the correctness of Wuhan to take timely isolation measures [6]. Wei et al. considered isolation and divided the infected population into light type, common type, heavy type and critical type, while a SEIR + CAQ model was established to predict the number of infected people with high performance of prediction results [7]. Zhou et al. considered the influence of isolation on the basis of the SEIR model, and compared the model with a logistic model, and concluded that rapid detection of cases and full implementation of quarantine are the keys to slowing down the pandemic situation [8]. They have carried out a series of useful and immediate research in such a pandemic of COVID-19.

However, most of the research has ignored people’s awareness and the impact of indirect infection on the spread of disease. For example, if people are fully aware of the harm of COVID-19, they will take the initiative to prevent it, such as reducing unnecessary going out, wearing masks, washing hands frequently and keeping distance with others. Indirect factors cannot be ignored in the spread of the disease. The motivation of this paper is to build a SEIR pandemic model that considers consciousness and indirect infection, and simulates the model and important parameters.

The risk assessment of work restoration during the pandemic is a multi-level comprehensive evaluation problem which involves many influencing factors. Some of these factors are qualitative and some are quantitative, so it is rather difficult to effectively assess risk of work restoration. At present, there are few studies on the risk assessment of work restoration during the pandemic, and there is a lack of guidance for enterprises returning to work. In this paper, based on the theory of infectious diseases and the previous analysis, a set of factors affecting the resumption of work were determined. Further, based on the concept of fuzzy comprehensive evaluation of multi-attribute entropy weight, a risk assessment model for enterprises to restore routine work during the pandemic is proposed, which can provide theoretical guidance for enterprises to make decisions.

The remainder of this paper is organized as follows. The general situation of the pandemic in China is given in Section 2. In Section 3, the consciousness-based SEIR model is built and important parameters are simulated. The potential risk assessment of work resumption in pandemic areas based on entropy fuzzy factor is given in Section 4. Finally, some conclusions are given in Section 5.

## 2. Overview of the Pandemic Transmission in China

**Figure 1:**
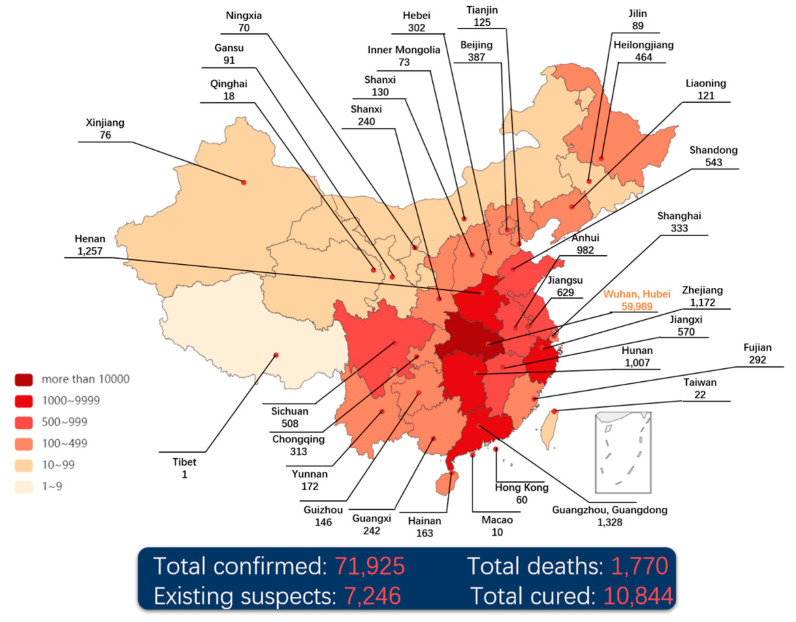
The heat map of coronavirus disease 2019 (COVID-19) in China (till 24:00 17 February 2020).

The daily confirmed data [9] released by the National Health Commission of the People’s Republic of China (NHC-PRC) is used to reproduce the spread of NCP through the heat map, shown in Figure 1. It is easy to find that the spread of the pandemic is mainly centered in Wuhan and spread to the surrounding areas aggressively. Through the population mobility, the epidemic has been spread to the central cities of China, i.e., Beijing, Shanghai, Guangzhou and other places, becoming the secondary transmission centers.

The NCP can be transmitted via human-to-human, droplets or direct/indirect contact [10,11]. There was no specific cure for the disease till then. On the other hand, traditional Chinese medicine has made certain progress in clinical treatment to alleviate the symptoms, and some experts pointed out that the plasma of cured patients has a positive therapeutic effect. As a consequence, COVID-19 is included in category-B infectious disease by NHC-PRC, which is a contagious disease subject to quarantine.

Wuhan is the most important city in central China and a transportation hub in China, and Wuhan has a large population with 11.2 million people. When the sudden outbreak of COVID-19 occurred on New Year’s Eve in China, it resulted in the rapid spread of the disease from Wuhan to the whole country, directly causing the difficulty of epidemic control. On the 23 January 2020, Wuhan city was locked down where all scheduled outbound traffic was suspended, a radical quarantine measure imposed by the local Government. A succession of actions have been taken to attempt to reduce the risk of infection and prevent the disease epidemic. Afterwards, traffic control has been implemented all over China, and temperature checkpoints have been set up at all highway junctions to check the vehicles. Furthermore, local governments implemented community closed management to restrict people travelling from the beginning of February 2020, especially in the Hubei area, and shut down the entertainment and shopping malls in succession. People are required to stay at home and not return to work but wait for further notice.

In the meantime, medical supplies and staff are deployed and arranged from all over the country to support Wuhan and Hubei Province. Besides, the first two makeshift hospitals, Leishenshan and Huoshenshan were constructed and completed in only 7 days ready to admit patients. Till midnight on the 17 February 2020, there were 72,436 confirmed cases, 1,868 death cases and 12,552 cured cases [9] (see Figure 1). A total of 92 confirmed cases were reported in Hong Kong, Macao and Taiwan, and 900 confirmed cases outside China, most of which are in countries near China. In particular, 542 confirmed cases were reported on the Diamond Princess cruise which was docked at the Japanese harbor.

**Figure 2:**
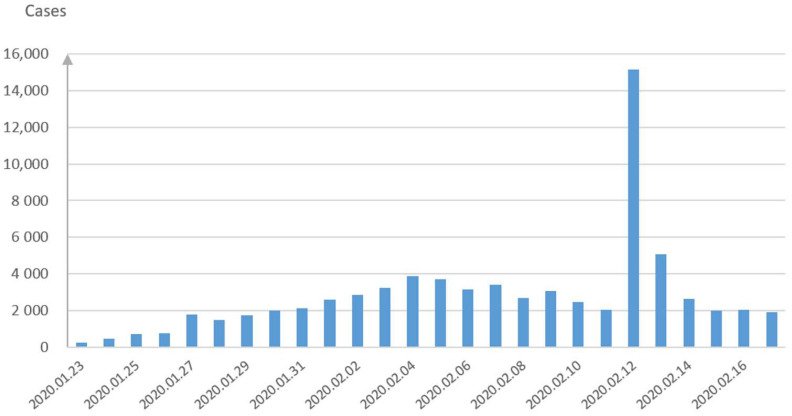
The daily new confirmed cases in China.

**Figure 3:**
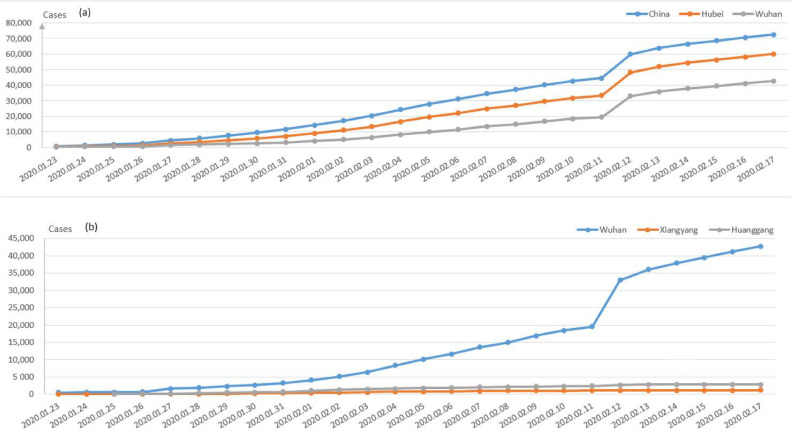
(**a**) Comparison of the cumulative confirmed cases of China, Hubei province and Wuhan; (**b**) comparison of the cumulative confirmed cases among three cities in Hubei province: Wuhan, Xiangyang and Huanggang.

Figure 2 demonstrates the confirmed cases on a daily basis during the late January and early February period in China. The cumulative confirmed cases in Wuhan city, Hubei Province and China are displayed in Figure 3a, while the comparison of the three main cities in Hubei is demonstrated in Figure 3b. Besides Wuhan city, Huanggang city and Xiangyang city are the most severe areas in Hubei Province. It can be seen from Figure 3b that the number of the confirmed cases in Wuhan is much larger than those in these two cities, which can also prove the effectiveness of decisively closing Wuhan traffic from the outside to stop the disease spread since 23 January 2020.

The data demonstrate that most of the confirmed cases are in Hubei Province, of which Wuhan is the most severely affected city. According to the reports, almost all confirmed cases outside Hubei province come from Hubei or have a history of contact with someone from Hubei. For instance, one of the authors who was infected NCP due to contact with people during a 114-people gathering (most people from Wuhan) on the 22 January 2020 has been just discharged from the hospital on the 16 February 2020.

**Figure 4:**
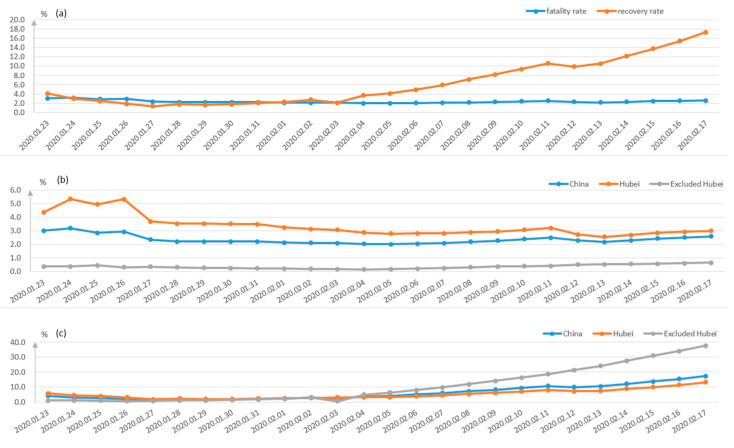
Fatality and recovery rates in different regions. (**a**) shows the comparison between the death rate and recovery rate in China, while (**b**,**c**) show the death rates and the recovery rates of China, Hubei and non-Hubei provinces, respectively.

**Figure 5:**
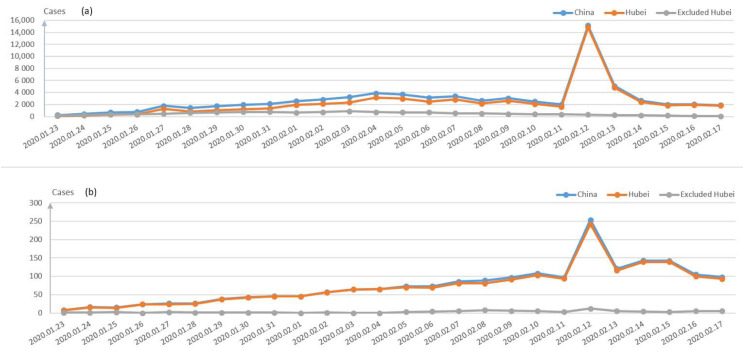
(**a**) The daily new confirmed cases in China, Hubei and non-Hubei provinces; (**b**) The daily new death cases of China, Hubei and non-Hubei province.

The fatality and recovery rates in different regions of China are depicted in Figure 4. It can be seen from Figure 4a that the recovery rate has increased rapidly since the 3 February 2020, while the death rate is rather stable. The trend of the death rate and recovery rate in Hubei and other areas are shown in Figure 4b,c, respectively. The death rate of Hubei Province is much higher than those of other Provinces, and the newly confirmed cases and dead cases are both much higher than the sum of other provinces, shown in Figure 5a,b.

As shown in Figure 5a, there was a sudden rise for the new confirmed cases in Hubei area on 12 February 2020, which was caused by the confirmation of the previous suspected population since the virus symptoms cannot be diagnosed at the first place [12,13]. It demonstrated that the density of the infected people in Hubei Province is still quite large, and the current medical facilities cannot meet the demand at the moment, so continuous medical facilities and staff were arranged in Wuhan, not to mention another 11 makeshift hospitals built to admit more patients. In contrast, the situation of the areas outside Hubei Province is easier to be control due to effective/timely community isolation and individual monitoring measures. Currently, different Apps in each district or even community on smart phones are developed to record personal travelling tracks when on the move, i.e., ‘Shen i’, ‘Health code’.

**Figure 6:**
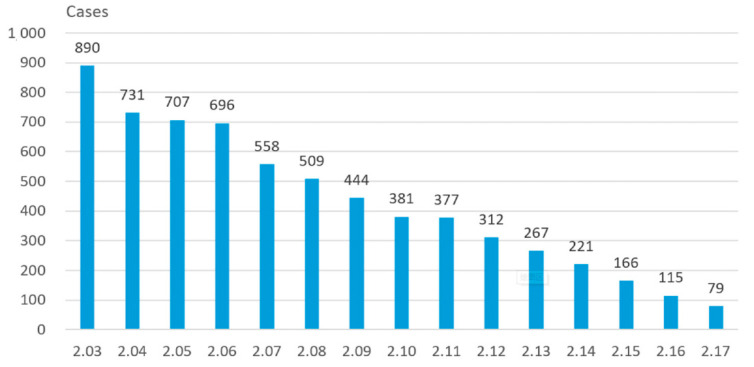
A diminishing trend of daily new cases in the non-Hubei area.

It is noted that the inflected population with confirmation was roughly stable after the 12th February 2020, due to appropriate traffic and medical support from other Provinces of China. The new cases from non-Hubei area are decreasing gradually, shown in Figure 6. Therefore, it can be concluded that the current measures taken by the Chinese Government can effectively prevent the spread of pandemic.

## 3. The Consciousness-Based SEIR Model for the Propagation of COVID-19

Considering the situation of indirect infection and the impact of the increase demand of medical resources on the spread of disease, the classical SEIR (Susceptible-Exposed-Infective-Recovered) model is improved with the introduction of consciousness. The involved factors are explained as—(1) the so-called consciousness, that is, through the media publicity, people have a certain understanding of the harm of the disease, so people will take different kinds of preventive measures on their own initiative, such as reducing unnecessary going out, wearing masks and washing hands frequently; (2) indirect infection, that is, the susceptible group is not infected by the latent or infected person, but by other means, i.e., the abandoned medical supplies, the latent or the objects carried disease (accident occurred at Beijing, e.g., package of frozen food) [14].

### 3.1. Model Formulation

Assume that (H1) each person has four possible states—Susceptible (S), Exposed (E), Infective (I) and Recovered (R); (H2) we introduce factors that can indirectly infect susceptible persons, such as discarded medical devices, objects touched by infected or latent persons, and the articles carrying pathogenic microorganisms. (H3) we introduce consciousness, and only susceptible people are divided into conscious and unconscious infections, and other states are not discriminated. (H4) After being cured, an infected individual turns into a convalescent person with disease resistance, but after a period of time, the disease resistance disappears and becomes susceptible once again. (H5) People who change from the recovery state to susceptible state have consciousness. (H6) The exposed (E) is also potentially infectious which could be more infectious than the infective (I) since they may be already infected but without any symptoms.

**Figure 7:**
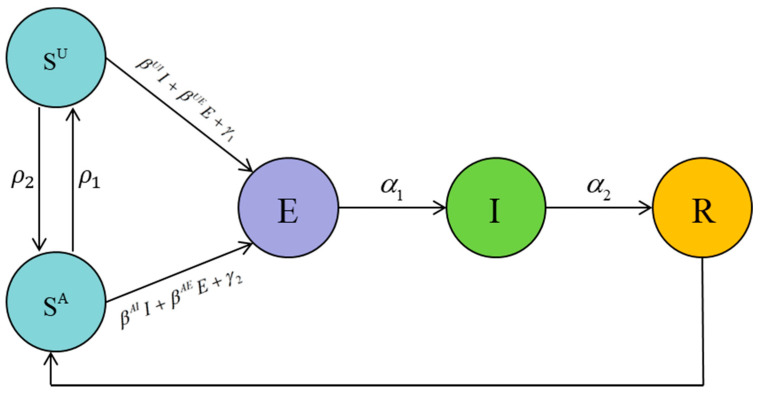
The schematic diagram of consciousness-based Susceptible-Exposed-Infective-Recovered (SEIR) model.

For simplicity, the variables and parameters in this consciousness-based SEIR model are summarized in Table 1 [15], and the schematic diagram of the mode is shown in Figure 7. $\rho_{1}$ represents the situation that along with the time, certain people would be relax and loose vigilance thus transferring from consciously susceptible to being unconsciously infected, while others will pay more attention to the disease. According to the hypothesis, we can get some conclusions:

(1) $$(1)\;\beta^{UI}>\beta^{AI};\;\;\;(2)\;\beta^{UE}>\beta^{AE};\;\;\;(3)\;\gamma_{1}<\gamma_{2}$$

Let *β^AI^* = *k* × *β^UI^*, *β^AE^* = *k* × *β^UE^*,$\gamma_{2}=k_{1}\times \gamma_{1}$, where 0 ≤ *k*, *k*_1_ ≤ 1. We get (a) when *k* = 0, which means that the susceptible person will not be infected after being conscious; (b) when *k* = 1, it means that susceptible people could be aware or unaware their health status, though the probability of the infection is the same.

Collecting the foregoing hypotheses, the new model can be represented by the difference equation as shown in Equation (2):

(2) $$\left\{ \begin{array}{l} S^{U}(t+1)=S^{U}(t)+\rho_{1}S^{A}(t)-\rho_{2}S^{U}(t)-rS^{U}(t)(\beta^{UI}I+\beta^{UE}E)/N-\gamma_{1}S^{U}(t)\\ S^{A}(t+1)=S^{A}(t)+\rho_{2}S^{U}(t)-\rho_{1}S^{A}(t)-rS^{A}(t)(\beta^{AI}I+\beta^{AE}E)/N-\gamma_{2}S^{A}(t)+\eta R(t)\\ E(t+1)=E(t)+rS^{U}(t)(\beta^{UI}I+\beta^{UE}E)/N+rS^{A}(t)(\beta^{AI}I+\beta^{AE}E)/N+\gamma_{1}S^{U}(t)+\gamma_{2}S^{A}(t)-\alpha_{1}E(t)\\ I(t+1)=I(t)+\alpha_{1}E(t)-\alpha_{2}I(t)\\ R(t+1)=R(t)+\alpha_{2}I(t)-\eta R(t) \end{array}\right.$$

with initial condition (*S^U^*(0), *S^A^*(0), *E*(0), *I*(0), *R*(0)) ∈ Ω, where Ω = {(*S^U^*(*t*), *S^A^*(*t*), *E*(*t*), *I*(*t*), *R*(*t*))|*S^U^*(*t*) + *S^A^*(*t*) + *E*(*t*) + *I*(*t*) + *R*(*t*) = *N*}, *N* is the total number of people.

### 3.2. Numerical Simulations

#### 3.2.1. Numerical Simulations

**Figure 8:**
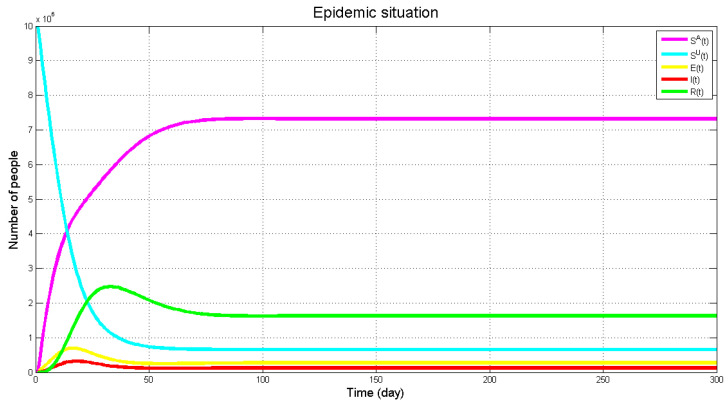
The evolution of the number of *E*(*t*), *I*(*t*) and *R*(*t*) along with time variation.

Matlab is used as the tool for numerical simulation. Under this consciousness-based SEIR model, the virus prediction result of the Wuhan areas is shown in Figure 8 with *k* = 0.6, *k*_1_ = 0.45, *r* = 1685, *β^UI^* = 0.04, *β^UE^* = 0.066, $\gamma_{1}$ = 0.006, *α*_1_ = 0.3, *α*_2_ = 0.65, η = 0.05, $\rho_{1}$ = 0.006, $\rho_{2}$ = 0.05. First, the numbers of the infected and asymptomatic infected people will arrive at the peak but numbers of *E* and *I* will decline due to the increase of treatment and attention priority. The recovery rate will reach its peak later (in accordance with reality), and decrease afterwards. In the meantime, *R* varies along with the tendencies of *E* and *I* but only with post-posed response.

As can be seen from the figure above, the population in each state has finally reached the equilibrium state, and the virus has always existed and will not disappear completely, which also indicates the necessity of long-term epidemic prevention. The number of infected and exposed people increased first then decreased afterwards, which may be due to the lack of effective prevention of the disease in the early stage, which led to the rapid spread of the disease nationwide. In the later stage, people took preventive measures against the disease, and the number of infected people began to decline.

#### 3.2.2. Impact of System Parameters

To learn more about this proposed model, we analyze the influence of several important parameters, namely $\rho_{1}$, $\rho_{2}$, *k*, $\gamma_{1}$, *α*_2_ on the number of exposed and infective people.

Let us look back at these parameters—*σ*_1_ means the probability of the conscious susceptible turning into the unconscious susceptible, *σ*_2_ means the probability of the unconscious susceptible turning into the conscious susceptible, *k* means the ratio of the unconscious infection rate to the conscious infection rate, $\gamma_{1}$ means the infection rate of the indirect factor to the unconscious susceptible person, and *α*_2_ means the probability of an infective person turns into a recovered person.

**Figure 9:**
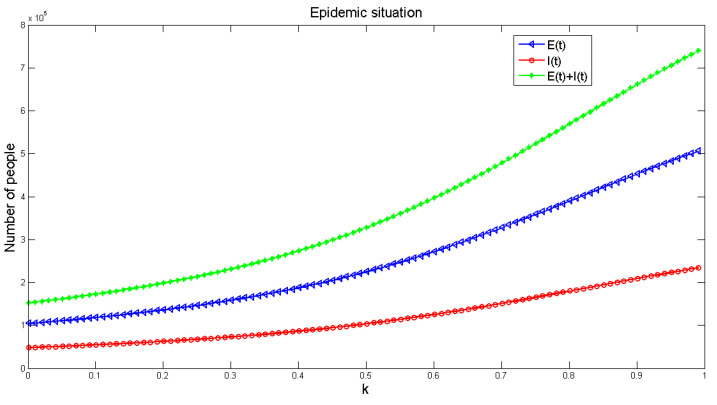
The effect of the ratio *k* on the infection number of *E*(*t*), *I*(*t*) and *R*(*t*) from the consciously susceptible to the unconsciously susceptible population.

The parameter values are the same as in Equation (1), and Figure 9 shows the influence of *k* on the number of diseases in the model. The graph indicates that the number of infected people increases with the increase of *k*. It is further noted that if *k* = 1, that is, without considering the influence of consciousness, the disease will be much larger. If we consider the influence of consciousness, the disease will be relatively small. Therefore, in order to reduce the spread of disease, it is helpful to strengthen the publicity of disease prevention and enhancing people’s awareness of the disease.

**Figure 10:**
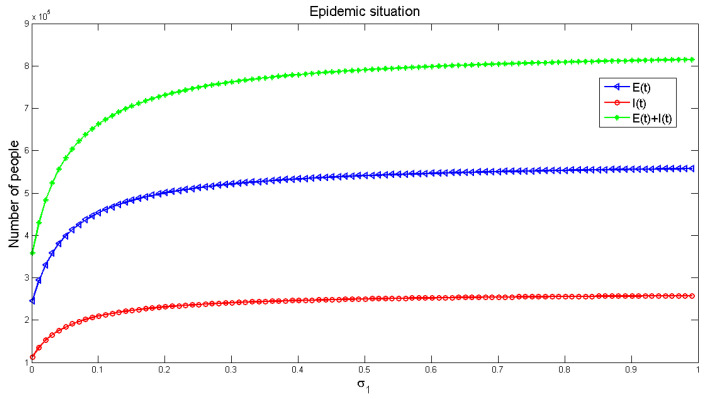
The influence of probability $\rho_{1}$ from conscious susceptible to unconscious susceptible on the number of infected people.

**Figure 11:**
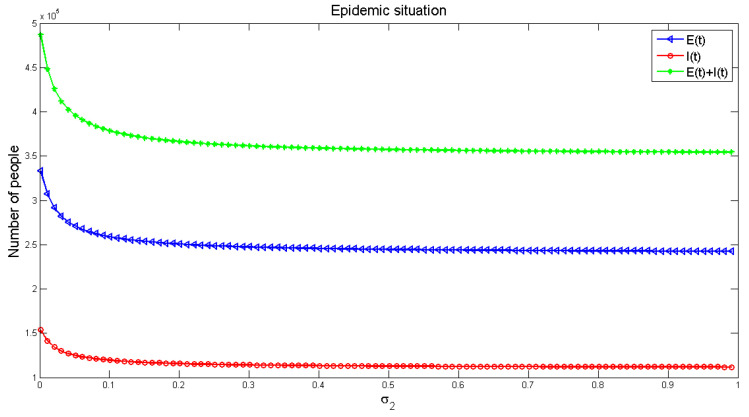
The influence of the probability $\rho_{2}$ from unconscious susceptible to conscious susceptible on the number of infected people.

Figure 10 and Figure 11 demonstrate the effects of $\rho_{1}$ and $\rho_{2}$ on the number of infected people, respectively. The number of infected people increases along with the increase of $\rho_{1}$ including the consciously susceptible and the unconsciously susceptible people, as shown in Figure 10. As can be seen in Figure 11, the number of infections decreases along with the increase of $\rho_{2}$ including the unconsciously susceptible and the consciously susceptible people. It also reflects that increasing people’s awareness about the disease is useful to reduce the spread of the disease.

**Figure 12:**
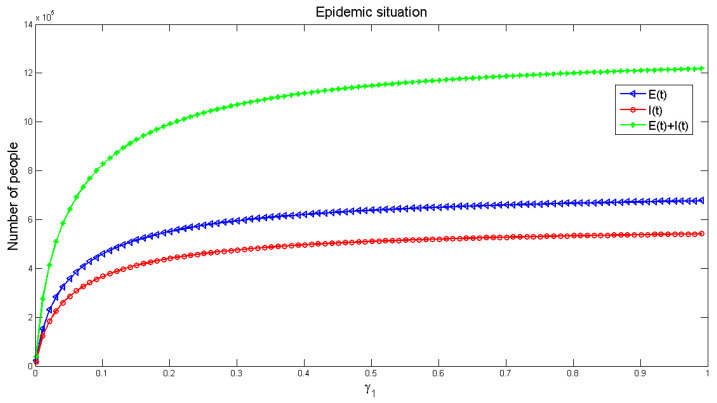
The influence of indirect infection factor *γ*_1_ on the number of infected people.

**Figure 13:**
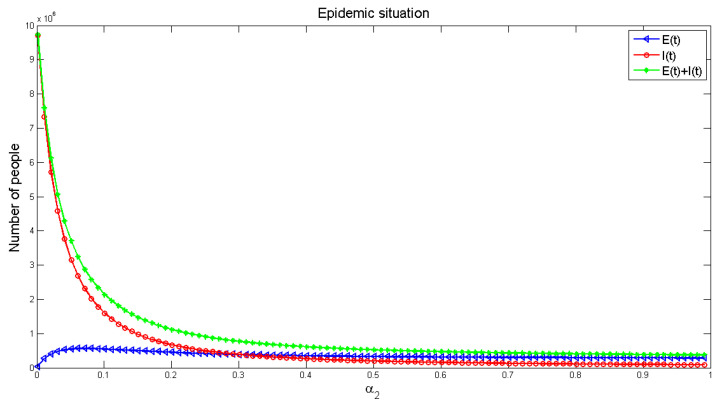
The effect of recovered rate *α*_2_ on the number of infected people.

Figure 12 and Figure 13 display the influence of the parameters on the number of infected people, respectively. It can be seen from Figure 12, with the increase of infection factors, the number of infected people is increasing, though it increases rapidly at the initial stage and gradually slows down in the later (roughly stable) stage. It shows that indirect infection has a great impact on the spread of the disease, and the early impact on the pandemic is severe. This requires that people in the early stage of the disease should block all possible indirect infection transmission routes, in order to reduce the number of infections. Figure 13 exhibits the number of infected people gradually decreased with the increase of recovery rate, and finally reached a stable level, but the number of infected people would not be reduced to zero. This indicates that strengthening medical measures and improving the cure rate of the disease can effectively reduce the number of infected people.

## 4. Potential Risk Assessment of Resumption of Work during Epidemic Period Based on Entropy-Fuzzy Factor

The assessment of the potential risk of resumption of work (RoW) during the COVID-19 epidemic period is a multi-level comprehensive evaluation problem, which involves various factors, especially influenced by certain subjective factors [16,17,18,19]. Therefore, it is necessary to distinguish the priority and determine the main factors. Based on the three basic components (i.e., source of infection, transmission route and susceptible population) of the epidemic of infectious diseases, the evaluation for the potential outbreak risk (EPOR) [20] of the factory RoW during the epidemic mainly includes such factors, listed in Table 2.

As shown in Table 2, each factor is composed of several subfactors, which are either qualitative or quantitative. However, the risk of work restoration during the epidemic can only be evaluated by the perfection degree. So, the fuzzy comprehensive evaluation method is used here, i.e., entropy weight combined with the fuzzy comprehensive analysis method [21]. The establishment process of the EPOR model is described as follows.

Step (1) Determine the impact factor set of the EPOR for RoW during epidemic.

The 1st-level indexes of the EPOR for rework during epidemic are composed of six primary factors (see the second column in Table 2), and the 2nd-level indexes are composed of 25 risk factors (see the third column in Table 2), written as *y* = {*y*_1_, *y*_2_, …, *y*_25_}.

Step (2) Determine the assessment set of EPOR for RoW during epidemic.

The assessment set is first determined by the experts, where the assessment of each factor can be divided into *m* levels, written as *V* = {*V*_1_, *V*_2_, …, *V_m_*}, denoting as {highest risk, higher risk, medium risk, low risk, lower risk}. Here, *m* = 5 is selected.

Step (3) Risk assessment based on entropy weight-fuzzy [22].

Let the fuzzy evaluation matrix for the EPOR for rework during epidemic be *R* = {*r_ij_*}*_n_*_×_ *_m_*, *i* = 1, 2, …, *n*; *j* = 1, 2, …, *m*, where *r_ij_* is the membership degree of the *j^th^*-level of the *i^th^* factor. The ratio of the *j^th^* level with the *i^th^* index based on the entropy weight can be calculated as:

(3) $$P_{ij}=(1+r_{ij})/\sum_{j=1}^{m}\left(1+r_{ij}\right)$$

Then the entropy of the *i^th^* index is calculated as:

(4) $$e_{i}=-\sum_{j=1}^{m}P_{ij}\times \ln P_{ij}/\ln m$$

So the entropy weight of the *i^th^* index can be calculated as:

(5) $$a_{i}=(1-e_{i})/\sum_{i=1}^{n}\left(1-e_{i}\right)$$

and the vector of the entropy weight of the EPOR of rework during epidemic can be written as *A* = (*a*_1_, *a*_2_, …, *a_n_*). Therefore, the corresponding comprehensive evaluation vector *B* is calculated as *B* = *A* × *R*, denoted as *B* = (*b*_1_, *b*_2_, …, *b_i_*, …, *b_m_*). There are many evaluation methods for the determination of *b_i_* considering the relationship among factors, such as principal subordinate relationship or balance all factors with the weighted average model [21].

### Case Study

According to the index listed in Table 2, a potential risk investigation for a randomly selected factory to restore work is conducted on the six primary indexes (*U_i_*, *i* = 1, …, 6), and the statistical results are shown in Table 3.

The evaluation matrix is calculated in detail with the following steps.

(1) The fuzzy evaluation matrix for obtaining the potential risk assessment of RoW is:

(6) $$R=\left[\begin{array}{ccccc} 0.08 & 0.17 & 0.22 & 0.29 & 0.24\\ 0.05 & 0.16 & 0.23 & 0.31 & 0.25\\ 0.06 & 0.18 & 0.31 & 0.21 & 0.24\\ 0.09 & 0.16 & 0.29 & 0.27 & 0.19\\ 0.06 & 0.18 & 0.27 & 0.32 & 0.17\\ 0.05 & 0.14 & 0.35 & 0.27 & 0.19 \end{array}\right]$$

(2) Calculate the ratio of the index of the *j^th^* level under the *i^th^* index based on Equation (3):

(7) $$P=\left\{\begin{array}{ccccc} 0.1800 & 0.1950 & 0.2033 & 0.2150 & 0.2067\\ 0.1750 & 0.1933 & 0.2050 & 0.2183 & 0.2083\\ 0.1767 & 0.1967 & 0.2183 & 0.2017 & 0.2067\\ 0.1877 & 0.1933 & 0.2150 & 0.2117 & 0.1983\\ 0.1767 & 0.1967 & 0.2117 & 0.2200 & 0.1950\\ 0.1750 & 0.1900 & 0.2250 & 0.2117 & 0.1983 \end{array}\right\}$$

(3) Calculate the entropy (*E*) of the *i^th^* index based on Equation (4):

*E* = {0.9989 0.9983 0.9985 0.9988 0.9983 0.9977} (8)

(4) Calculate the entropy weight of the *i^th^* index according to Equation (5):

*A* = {0.1162 0.1818 0.1548 0.1217 0.1829 0.2427} (9)

(5) Calculate the fuzzy comprehensive evaluation using the weighted average model [2]:

*B* = *A* × *R* = {0.0900 0.1800 0.2427 0.2427 0.1900} (10)

Here, the risk assessment vector is set as *V* = {0.95, 0.75, 0.5, 0.35, 0.1}, so the comprehensive evaluation score of the potential risk assessment of a factory to restore work is calculated as *D* = *B* × *V*, thus the potential risk of resuming work at this factory is calculated to be 0.4423. In other words, the probability of the potential risk of this factory returning to work is about 44.23%. Therefore, the head of the Enterprises can take necessary measures to restore normal production step by step.

## 5. Conclusions

In this paper, the dealt measures and public awareness of COVID-19 are analyzed and the outbreak of COVID-19 in the late January and early February in China is overviewed where the local government took appropriate measures to stop a severe pandemic. Further, a modified consciousness-based SEIR model is provided to describe the disease propagation, targeting the main factors affecting the spread of the COVID-19. This paper focused on how people should tackle this pandemic for work resumption with appropriate public awareness including consciously or unconsciously knowing the infective status. The entropy and fuzzy comprehensive evaluation methods are combined to establish a potential risk assessment model for large-scale work resumption, so that such qualitative and fuzzy relationships related to the epidemic can be solved mathematically. As a result, the risk of work resumption under different protection conditions are evaluated by the model accurately and scientifically, which can provide theoretical guidance for the decision-making of the government and enterprises.

## Figures and Tables

**Table 1:** Description of the involved parameters.

**Variables**	**Description**
*S^U^*(*t*)	The number of unconscious susceptible people at time *t*.
*S^A^*(*t*)	The number of conscious susceptible people at time *t*.
*E*(*t*)	The number of exposed people at time *t*.
*I*(*t*)	The number of people infected at time *t*.
*R*(*t*)	The number of people recovered at time *t*.
**Parameters**	**Description**
*k*	The proportional coefficient between *β^AI^* and *β^UI^* & *β^AE^* and *β^UE^*.
*k*_1_	The proportional coefficient between *γ*_1_ and *γ*_2_.
*r*	The number of contacts per person in *t* time interval.
*β^AI^*	The probability that a conscious susceptible person is infected by an infected person.
*β^AE^*	The probability that a conscious susceptible person is infected by an exposed person.
*β^UI^*	The probability that an unconscious susceptible person is infected by an infected person.
*β^UE^*	The probability that an unconscious susceptible person is infected by an exposed person.
$\rho_{1}$	The probability that a conscious susceptible person turn into an unconscious susceptible person.
$\rho_{2}$	The probability that an unconscious susceptible person turn into a conscious susceptible person.
*γ*_1_	The infective rate of indirect factors to the unconscious susceptible people.
*γ*_2_	The infective rate of indirect factors to the conscious susceptible people.
*α*_1_	The probability of an exposed person turns into an infective person.
*α*_2_	The probability of an infective person turns into a recovered person.
η	The probability that a recovered person loses immunity into a conscious susceptible person.

**Table 2:** The evaluation for the potential outbreak risk (EPOR) of work resumption during epidemic.

Index	Primary Factors (1st-Level)	Subfactors (2nd-Level)
*U*_1_	Self-protection	Wear disposable surgical masks; Wear KN95 masks; Separate eating;Wear KN95 masks and goggles; Wear Protective clothes; Wash hands
*U*_2_	Company prevention	Quarantine measure; Epidemic prevention; Monitoring measure; Off-peak commuting; Perfect disinfection; Harmless treatment
*U*_3_	Protection supervision level	Perfecting epidemic prevention supervision system; Perfecting of quarantine supervision system
*U*_4_	Production management level	Density of workers at workplace; Worker communication frequency;Plant sector density; Management awareness of prevention and control
*U*_5_	Internal and external outbreak threat	Severity of the outside; External isolation strength; Epidemic trend
*U*_6_	Environmental impact	Traffic condition; River abundance density; Bad weather;Mobility of contaminated objects

**Table 3:** Statistical result for major risk evaluation of RoW.

The 1st-Level Index		Risk Assessment Set			
	Highest (*V*_1_)	Higher (*V*_2_)	Medium (*V*_3_)	Low (*V*_4_)	Lower (*V*_5_)
*U*_1_	8%	17%	22%	29%	24%
*U*_2_	5%	16%	23%	31%	25%
*U*_3_	6%	18%	31%	21%	24%
*U*_4_	9%	16%	29%	27%	19%
*U*_5_	6%	18%	27%	32%	17%
*U*_6_	5%	14%	35%	27%	19%

## Data Availability

The data that support the findings of this study are collected from the National Health Commission of the People’s Republic of China, available from the corresponding author, upon reasonable request.
